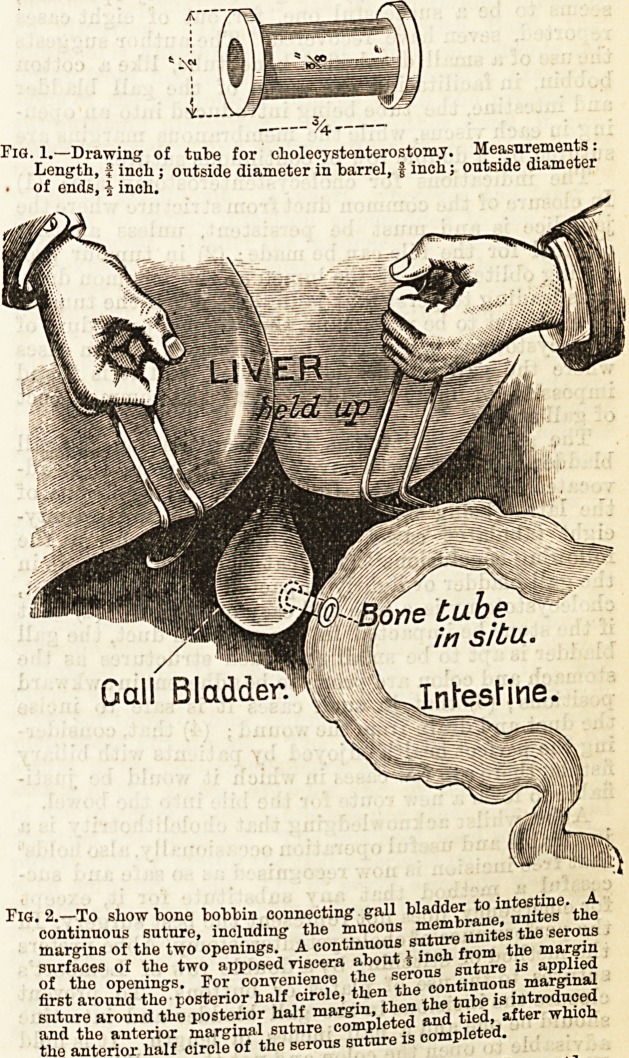# Liver and Gall Bladder

**Published:** 1894-01-20

**Authors:** 


### MEDICAL PROGRESS.

### LIVER AND GALL BLADDER.

As a result of the incarceration of a stone a number of diseases affecting both the liver and the biliary passages may be produced, and these are classified[1] by Dr. Parker as (1) cholangitis, (2) cholecystitis, (3) hepatitis, (4) pericholangitic inflammations and neoplasms, (5) pericholecystitic inflammations and neoplasms. Mayo Robson, who has operated on more than fifty cases, mentions[2] the following complications and dangers of cholelithiasis which have come under notice: Repeated attacks of biliary colic, so-called "spasms," without jaundice; biliary colic with persistent jaundice and its consequences, such as hæmorrhage; intermittent pyrexia, with jaundice and pain; persistent vomiting, with such serious digestive disturbances as to threaten death from inanition or exhaustion; acute intestinal obstruction due to impaction of a large gall stone in the bowel, or to peritonitis; stimulation of intestinal obstruction due to irritation and pain; localised peritonitis, with or with out ulceration of the bile passages; perforative peritonitis; septicæmia due to ulceration of bile passages; abscess of liver; empyæma of gall bladder; dropsy of gall bladder; abscess of abdominal walls; pyelitis of right kidney, and collapse due to intense pain.

Where there is neither jaundice nor distension of the gall bladder, and when so-called "spasms" are frequently recurring and do not yield to medical treatment, the gall stones will usually be found in a shrunken gall bladder or in the cystic duct, but where jaundice is present the stones will probably be found in the common duct; and he has in either of these cases almost invariably found numerous and firm adhesions, showing that the attacks have been frequently associated with local peritonitis. Where there is distension of the gall bladder, associated with pain, but without jaundice, one large gall stone or several smaller ones will probably be found blocking the neck of the gall bladder and the cystic duct. When there is persistent jaundice, with distension of the gall bladder and without marked pain, malignant disease is to be suspected, especially if there is an absence of the intermittent pyrexia which usually co-exists with the presence of gall stones in the common duct. The enlargement of the gall bladder in these cases is worth noting, as in all cases of malignant disease with jaundice—on which Robson has operated—the enlarged gall bladder has formed a perceptible tumour. For diagnostic purposes he advises to make a small exploratory incision, then to empty the gall bladder by the aspirator, and afterwards to explore the bile passages with the fingers.

Cholecystotomy is the operation *par excellence* in the treatment of gall stones, and although it is difficult when adhesions are present, the risk is small in the absence of malignant disease and persistent jaundice. Thirty such cases all recovered; and out of fifteen other cholecystotomies for jaundice with gall stones in the absence of cancer, Robson has not lost one patient as a result of the operation.

To avert the danger from hæmorrhage in cholæmic cases, the author supports the use of chloride of calcium in fifteen grain doses every four hours for two days before operation. Temporary drainage of the gall bladder is preferred to immediate suture. Instead of suturing the edges of the incision in the gall bladder to the skin, Robson fixes it to the aponeurotic layer of the abdominal wall, and thus lessens the danger of a fistula. Where the gall bladder is shrunken and cannot be brought to the surface, it is often possible to tuck the parietal peritoneum down and suture it to the margins of the incision in the viscus, but where this cannot be done the omentum is utilised, suturing it to the gall bladder and to the parietal peritoneum, thus occluding the peritoneal cavity. Where occlusion in this way cannot be effected, the insertion of a drainage tube into the gall bladder without suture of the margins to the wound seems to be efficient. If common or cystic ducts cannot be cleared of stones by fingers or forceps, then cholelithotrity may be attempted. Robson first tries to crush them between the finger and thumb, and failing this, employs forceps covered with indiarubber. If this method fails, incision of the

FIG. 1.—Drawing of tube for cholecystenterostomy. Measurements: Length, $\frac{3}{4}$ inch; outside diameter in barrel, $\frac{3}{8}$ inch; outside diameter of ends, $\frac{1}{2}$ inch.

FIG. 2.—To show bone bobbin connecting gall bladder to intestine. A continuous suture, including the mucous membrane, unites the margins of the two openings. A continuous suture unites the serous surfaces of the two apposed viscera about $\frac{1}{3}$ inch from the margin of the openings. For convenience the serous suture is applied first around the posterior half circle, then the continuous marginal suture around the posterior half margin, then the tube is introduced and the anterior marginal suture completed and tied, after which the anterior half circle of the serous suture is completed.

duct and removal of the concretion may be done, the opening in the duct being sutured and the right kidney pouch drained.

The following conditions are indications for cholecystotomy: (1) In frequently-recurring biliary colic without jaundice, where medical treatment has failed; (2) in persistent jaundice, where the onset was ushered in with pain, and where recurring pains, with or without ague-like attacks, render it probable that the cause is gall stones in the common duct; (3) in distended gall bladder from impaction of calculi in the ducts; (4) in empyæma of the gall bladder; (5) in persistent jaundice with enlargement of the gall bladder dependent on some obstruction in the common duct, even where the cause cannot be clearly made out; but in such cases the increased risk should be borne in mind, as malignant disease may not improbably be the cause of the obstruction.

*Cholecystectomy.*—Robson has had three cases, in all of which recovery ensued. The operation is not difficult, and in his last case a single fine silk ligature around the cystic duct answered quite as well as the more complicated procedures. The following conditions are indications for cholecystectomy: (1) Where, after cholecystotomy, a mucous fistula persists, dependent on stricture of the cystic duct; (2) where, under similar circumstances, owing to accumulation of fluid in the gall bladder, the pain recurs as soon as the fistula has closed; (3) in cancer, if the disease be limited to the gall bladder: wherever there is obstructive jaundice, cholecystectomy is contraindicated.

Cholecystenterostomy, though not an easy operation, seems to be a successful one, for, out of eight cases reported, seven have recovered. The author suggests the use of a small decalcified bone tube, like a cotton bobbin, in facilitating the union of the gall bladder and intestine, the tube being introduced into an opening in each viscus, while the membranous margins are sutured by a double row of continuous suture.

The indications for cholecystenterostomy are: (1). In closure of the common duct from stricture where the jaundice is and must be persistent, unless another channel for the bile can be made; (2) in tumour producing obliteration of the lumen of the common duct, thus leading to persistent jaundice; but if the tumour be made out to be malignant, the simpler procedure of cholecystotomy had better be performed; (3) in cases where the gall bladder is distended, and it is found impossible or impracticable to clear the common duct of gall stones.

The general indications for operations on the gall bladder here set forth have also in the main been advocated[3] by Czerny and Ignatow[4], the observations of the latter being based on two hundred and seventy-eight tabulated cases. Dr. Duncan has come to the following conclusions[5]: (1) That when the stones lie in the gall bladder or lightly impacted in the cystic duct, cholecystotomy is a safe and easy operation; (2) that if the stone be impacted in the common duct, the gall bladder is apt to be small, and such structures as the stomach and colon are prone to be adherent in awkward positions; (3) that in such cases it is safe to incise the duct and drain from the wound; (4) that, considering the perfect health enjoyed by patients with biliary fistula, there are few cases in which it would be justifiable to form a new route for the bile into the bowel.

Abbe, whilst acknowledging that cholelithotrity is a justifiable and useful operation occasionally, also holds[6] that free incision is now recognised as so safe and successful a method that any substitute for it, except for emergency, does not represent the best surgery. In the performance of cholecystenterostomy, Abbe prefers the method of stitching by a double row of Lembert's sutures to the use of plates or buttons. To prevent closure the incisions in the gall bladder and intestine should be one and a half inches in length. It is held advisable to open the colon and not the small intestine. The bile is not now considered to be a necessary digestive secretion, and the subjects of chronic external fistula pouring out the entire bile flow often gain weight and health. Dr. Cabot says[7] that in a case where the calculus was impacted in the cystic duct he found a wire loop passed beyond the calculus the readiest means of withdrawing it, the side of the loop next the stone being rough and sharp so as to hold it. Terrier, speaking on the subject of choledochostomy, thinks that we must reserve our judgment on this operation. He has collected[8] four cases, all fatal, and points out that in two of these cases the distension of the bile duct, though clearly due to obstruction, was not associated with lithiasis. In each instance the gall bladder was much shrunken, and its walls sclerosed and surrounded by cicatricial tissue. The fatality is probably due to the fact that extreme distension of the bile duct is often accompanied by infection of the biliary passages.

In the treatment of hydatids of the liver, Mr. Clutton rejects[9] operation by aspiration, electrolysis, or the introduction and retention of a large trocar, and prefers abdominal or thoracic section according to the position of the cyst. He would operate in the usual method by two stages, except (*a*) when the cyst is partially adherent, and (*b*) when the cyst is immediately below the surface and can be easily evacuated. In the latter case his reason for operating by one stage, instead of two, is because he does not think it desirable to risk its rupture in another part in the interval between the first and second operation. Bruce Clarke is also similarly dissatisfied with the ultimate results of aspiration, and proposes[10] that after the abdomen has been opened in the ordinary way, the cyst wall should be peeled off from the surrounding tissues and removed. The edges of the cavity are stitched to the abdominal wound and drained. He reports three cases of hydatid of the liver, and one of hydatid of the kidney, treated in this way; all of them recovered.

Partial resections of the liver have now been recorded in several instances. Schmidt reports[11] a successful case of extirpation of a gummatous portion of the liver. The tumour was situated in the right hypochondriac region, and moved with the diaphragm. It was supposed to be connected with the transverse colon and abdominal section was performed for its removal. It was, however, found to be attached to the lower quadrant of the left lobe of the liver. It was about as large as an egg and somewhat lobulated. There were no adhesions, and no other nodules were found in the liver. The tumour was brought out through the abdominal wound; the peritoneum surrounding its base was secured by a circular stitch to the parietal peritoneum, and the base of the neoplasm was surrounded by an elastic ligature, and was cut through. Four arteries and six veins were ligatured, and a point of oozing was touched with the thermo-cautery. The extra peritoneal position of the wound was secured by a second row of sutures, including the skin and the walls of the liver, and the dressing was completed by iodoform packing. Microscopic examination proved the growth to be a gumma. The patient recovered without complications. Von Bergmann has in a similar way removed an adenoma of the liver, and Bardeleben has recently stated[12] that he had removed a sarcoma of the liver two years ago, and there has been no recurrence as yet. Koenig also stated[13] that recently he had removed hepatic tumours in several cases. In one the tumour was removed by making a circular incision around the pedicle, and the adjacent peritoneum lifted up; the tumour was then removed, and the peritoneum sutured over the base. This proceeding, according to Koenig, stops all hæmorrhage from the pedicle, and is to be preferred in place of plugging with a gauze tampon.

[1] Annals of Surgery, June, 1893. [2] Brit. Med. Journ., April 15, 1893. [3] Dent Med Work, No. 23, 1892, and Brit. Med. Journ., Jan. 7, 1893. [4] Quoted in Med. Rec., N.Y., Aug. 19, 1893. [5] Edin. Med. Journ., June, 1893. [6] Med. Rec., N.Y., May 6, 1893. [7] Boston Med. and Surg. Reporter, Dec. 8, 1892. [8] Rev. de Chir, Feb., 1893, and Brit. Med. Journ., April 8, 1893. [9] Clinical Journal, June 21, 1893. [10] Brit. Med. Journ., April 1, 1893. [11] Dent. Med. Work, No. 8, 1893, and Therapeutic Gazette, April 15. 1893. [12] Brit. Med. Journ., May 27, 1893. [13] Op. Cit.

## Figures and Tables

**Fig. 1. Fig. 2. f1:**